# Cellular Therapeutic Approaches to Cytomegalovirus Infection Following Allogeneic Stem Cell Transplantation

**DOI:** 10.3389/fimmu.2020.01694

**Published:** 2020-07-31

**Authors:** Manar S. Shafat, Vedika Mehra, Karl S. Peggs, Claire Roddie

**Affiliations:** ^1^Research Department of Haematology, UCL Cancer Institute, University College London, Cancer Institute, London, United Kingdom; ^2^Department of Haematology, University College London Hospitals NHS Foundation Trust, London, United Kingdom

**Keywords:** cytomegalovirus, virus-specific T cells, cellular therapies, antiviral therapy, infection

## Abstract

Cytomegalovirus (CMV) infection is common following allogeneic hematopoietic stem cell transplant (HSCT) and is a major cause of morbidity and increased mortality. Whilst pharmacotherapy can be effective in the prevention and treatment of CMV, these agents are often expensive, toxic and in some cases ineffective due to viral resistance mechanisms. Immunotherapeutic approaches are compelling and early clinical trials of adoptively transferred donor-derived virus-specific T (VST) cells against CMV have demonstrated efficacy. However, significant logistical challenges limit their broad application. Strategies to optimize VST manufacture and cell banking alongside scientific developments to enhance efficacy whilst minimizing toxicity are ongoing. This review will discuss the development of CMV-specific T-cell therapies, the challenges of widespread delivery of VSTs for CMV and explore how VST therapy can change outcomes in CMV infection following HSCT.

## Introduction

Cytomegalovirus (CMV) infection following allogeneic hematopoietic stem cell transplantation (HSCT) is a major cause of morbidity and mortality. Early clinical trials demonstrate that adoptive transfer of donor-derived virus-specific T cells to restore virus-specific immunity is an effective strategy to control CMV infection after HSCT, conferring protection in 70–90% of patients (1). The field has evolved rapidly to develop solutions to some of the CMV cell therapy manufacturing challenges identified in early clinical studies and to define strategies to deliver CMV cell therapy to patients with virus-naive donors. This review discusses the seminal early studies and explores cutting-edge novel technologies that broaden the feasibility and the scope of virus-specific T cells for at risk patients.

## Biology of CMV

Cytomegalovirus (CMV) is the fifth member of the herpes family of viruses. Structurally, it consists of an icosahedral capsid with an immunogenic glycoprotein B enriched envelope and tegument with abundant pp150 (UL32) and pp65 (UL83) proteins, essential for virus maturation, cellular entry and spread and a 230-kb double-stranded linear DNA genome (2). During the infective phase, three subgroups of viral proteins are rapidly synthesized: immediate-early (IE), early (E), and late (L). Within a few hours of viral entry, the IE proteins are generated and act as transcriptional activators of the CMV early (E) genes which encode proteins such as the UL55, UL95, and UL97 protein kinases. UL97 can phosphorylate antiviral drugs such as ganciclovir and mutations can lead to viral drug resistance. Twenty-four to forty-eight hours post-infection, later (L) genes express proteins that play a role in the structural formation of the CMV virion. Some of these genes may be transcribed at the early stage but are only translated after DNA replication.

T-cell responses to CMV in healthy individuals is heterogeneous. In one study, overlapping 15-mer peptides derived from 213 CMV open reading frames (ORFs) were administered to 33 healthy volunteers and immune reactivity to 151 of the 213 ORFs was subsequently demonstrated. This suggests that there is a broad range of CMV-specific targets that can be recognized by healthy T-cells (3). However, the tegument protein UL83/pp65 is widely accepted as the major immune-dominant target of CMV-specific T-cell responses. Unlike IE proteins, pp65 does not depend on viral genome expression during CMV infection and can be found in abundance on the cell surface. High numbers of circulating pp65 specific cytotoxic T lymphocytes (CTLs) have been observed in infected individuals (4) and analysis of pp65-specific T cells has shown that some donors have a refined response, recognizing only a single peptide, whereas others can recognize multiple peptides in the pp65 gene product (5).

CMV can infect many cell types including leukocytes and endothelial cells and has a replication cycle of ~1 day in the naïve host. CMV infection initially triggers a proinflammatory response characterized by secretion of acute phase proteins and type 1 cytokines (including interleukin-18 and Interferon-γ) by the innate immune system followed by adaptive humoral and cell-mediated adaptive immune responses (6). Neutralizing antibodies to envelope glycoprotein B are observed, but humoral responses are thought to confer limited protection overall. Rather, CMV specific T-cells are felt to be critical to recovery from CMV infection and T-cell subset analysis indicates that ~10% of all memory CD4+ and CD8+ T cells compartments are directed against CMV during active infection (3). Natural killer cells also play a role in the early control of CMV infection, Indeed, studies show that impaired NK function can lead to heightened susceptibility and more severe infections with herpesviruses (7).

Despite this scale of host immune response, CMV is never eliminated from the immunocompetent individual, as it expresses immune evading genes that restrict innate and adaptive immune responses. As a result, CMV persists in a state of latency following primary infection (8) and can periodically reactivate during the host's lifetime (5).

## Risks for CMV Infection in the Immunocompromised Host

CMV reactivation progressing to CMV disease is prevented in immunocompetent hosts by the innate and adaptive immune systems. By contrast, where patients are immunocompromised, such as in the setting of HSCT, CMV infection or reactivation can proceed unhindered, leading to clinical disease, life-threatening end organ damage (pneumonitis, retinitis, colitis, and hepatitis) and heightened mortality (9). Reactivation of CMV occurs in 30–60% of CMV seropositive recipients and in 10–30% of seronegative recipients receiving stem cells from seropositive donors (10). In some early reports, the mortality associated with CMV was as high as 25% in CMV seropositive HSCT recipients (11). The risk of CMV reactivation relates to both the conditioning chemotherapy delivered pre-HSCT to ablate/suppress the host immune system prior to transfer of donor stem cells and the use of immunosuppressive agents employed routinely to prevent graft vs. host disease (GvHD) (12). Myeloablative conditioning regimens incorporating total body irradiation (TBI) confer a higher risk of CMV reactivation. Further, T-cell depleting therapies such as anti-thymocyte globulin (ATG), fludarabine, tyrosine kinase inhibitors (TKI) such as dasatinib and the use of *ex-vivo* T-cell depleted stem cells also increase the risk of CMV reactivation and infection (13–15).

Prevention and management of GvHD using pharmacological immunosuppression (including corticosteroid therapy) is a major risk factor for CMV reactivation. It is difficult to quantitate absolute risks, but lymphopenia with low absolute CD4+ T-cell counts and undetectable CMV-reactive CD8+ T-cells are thought to be contributory (16–20).

## Pharmacotherapy for CMV Reactivation/Infection in the Post-HSCT Setting

CMV reactivation is common in the early post-HSCT setting. For this reason, virologic surveillance of the blood for CMV by quantitative polymerase chain reaction (qPCR) during the first 100 days post-HSCT is critical (21, 22). Two main strategies are employed for the management of CMV reactivation to prevent CMV disease: (1) pre-emptive treatment and (2) universal CMV prophylaxis (23). Pre-emptive antiviral pharmacotherapy is commonly used in asymptomatic patients with rising CMV DNA titers in the blood and continued until the blood viral load is undetectable. This has been shown to reduce the incidence of early CMV disease from 30 to <5% (24) but to date has not demonstrated an overt correlation with overall survival (22, 25, 26). Commonly used antiviral pharmacotherapies for pre-emptive treatment include Ganciclovir, its prodrug Valganciclovir, Foscarnet, and Cidofovir (27, 28). Ganciclovir is administered intravenously and undergoes phosphorylation to ganciclovir-triphosphate which is an inhibitor of viral replication. Valganciclovir has the same mechanism of action, but with a 10-fold higher bioavailability. Foscarnet, a pyrophosphate analog works by inhibiting viral kinases essential for replication. It is administered for treatment of ganciclovir-resistant CMV and also when cytopenias preclude ganciclovir. Cidofovir (and the related agent Brincidofovir) are nucleotide analogs of cytosine that incorporate into viral DNA and disrupt viral replication. Brincidofovir has a higher bioavailability than cidofovir and does not act as an organic anion transporter substrate, making it significantly less nephrotoxic.

Historically, prophylactic antiviral pharmacotherapy for CMV has been limited by the commonly observed toxicities associated with treatment (29–31). However, uptake of potentially less toxic novel agents in this space is gaining traction. Maribavir, a UL97 protein kinase inhibitor, is currently being evaluated in the pre-emptive space in a Phase III randomized study (against valganciclovir) and in the refractory viraemia setting (against Foscavir) (27). Maribavir has also been tested as CMV prophylaxis in a phase II study and results suggest a reduced incidence of CMV reactivation in the first 100 days following HSCT with a tolerable toxicity profile (32). Unfortunately, this signal was not borne out in a placebo-controlled phase III study where Maribavir failed to prevent CMV disease (33). In contrast, an important study looking at the use of prophylactic Letermovir administered over the first 100 days post-HSCT revealed a significantly lower risk of clinically significant CMV infection compared with placebo and an acceptable safety profile (34). Letermovir works by inhibiting CMV replication by binding to the viral terminase complex (34). Real world data on Letermovir in the setting of primary and secondary prophylaxis indicate that this exciting new agent may represent a new gold standard in CMV prevention for high risk patients (35–37). As such, it has been granted orphan designation by the European Medicines Agency (EMA) and the United States (US) Food and Drug Administration (FDA).

Despite significant advances in antiviral pharmacotherapies, several significant limitations remain. Drug toxicity (including myelosuppression leading to bacterial and fungal infection, and nephrotoxicity) and antiviral drug resistance mechanisms are common and can compromise the delivery and efficacy of both prophylactic and pre-emptive drug approaches (29, 30, 38). Resistance to ganciclovir can occur due to prolonged drug exposure and is due to altered expression/activity/mutation of the pUL97 and pUL54 viral kinases. Drug resistant CMV disease is observed in patients with poor clinical and virologic responses to treatment, typically, where the viral load increases for more than 14 days despite therapy. If resistance is suspected, genotyping and drug switch is recommended but in critically ill patients the prognosis is bleak and novel therapies are required (39). It is also recognized that upon cessation of prophylactic therapy, there is a real risk of delayed CMV reactivation. Subgroup analysis within the Letermovir study suggests that patients with HLA-mismatched donors, cord blood donors, T-cell depleted grafts and those with GvHD requiring immunosuppression are all at high risk of reactivation upon drug cessation (36).

## Cellular Immunotherapy for CMV Reactivation/Infection in the Post-HSCT Setting

The limitations associated with CMV pharmacotherapeutics and the ongoing morbidity and mortality associated with CMV infection and reactivation in patients post-HSCT prompts ongoing research efforts in the cellular immunotherapy space. Indeed, trials have shown that adoptive transfer of virus-specific cytotoxic T lymphocytes (VSTs) can rapidly reconstitute antiviral immunity post-HSCT (Table 1).

**Table 1:** CMV-directed T-cell immunotherapy trials.

**Year of study**	**Cell therapy**	**Number of patients**	**CMV-specific T-cell generation**	**Dose/Kg bw**	**CMV-related outcome**	**GvHD status**	**References**
**MATCHED DONOR CMV T-CELL CLONES**							
1995	CD8 T-cell clones	14	Autologous fibroblasts infected with CMV AD196 strain	Dose escalation range 33 × 10^6^ to 1 × 10^9^/m^2^	14/14 patients cleared CMV disease	GvHD grade I-II, *n* = 3	(40)
**MATCHED DONOR CMV T-CELL LINES (USING PEPTIDE/PROTEIN PULSED FEEDER CELLS)**							
2002	CMV-specific polyclonal T-cells	8	Autologous irradiated feeder cells pulsed with CMV antigen	1 × 10^7^/m^2^	5/8 cleared after first dose, 1/8 cleared after dose 2. 1/8 did not clear, 1/8 non-evaluable	None	(41)
2003	CMV-specific polyclonal T-cells	16	Autologous DC feeder cells pulsed with CMV antigen	1 × 10^5^/kg	8/16 cleared CMV infection without antiviral therapy, 2/16 had viral reactivation	Cutaneous GvHD grade I, *n* = 3	(42)
2005	CMV-specific CD4+ T-cells	25	MRC-5 feeder cells infected with CMV lysate	1 × 10^5^-1 × 10^6^/kg	7/25 had CMV reactivation, 5/25 had CMV disease out of which 2 died	GvHD grade II *n* = 1	(43)
2012	CMV-specific polyclonal T-cells	7	Autologous-derived cells pulsed with pp65 and/or IE1 peptide	2.5 × 10^5^ to 5 × 10^5^ CD3+ CMV T-cells/kg	5/7 has CMV-specific T-cell activity, 2/7 did not have response	None	(44)
2015	CMV-pp65 polyclonal T-cells	16	Stimulated with autologous cytokine-activated monocytes with CMV pp65 protein	5 × 10^5^/kg × 1 dose to 1 × 10^6^/kg × 3 weekly dose	14/16 cleared viremia	None	(45)
**MATCHED DONOR CMV T-CELL LINES (USING GENE ENGINEERED FEEDER CELLS)**							
2006	CMV, EBV and Adenovirus (Adv) specific CD4+ and CD8+ polyclonal T-cells	11	HSCT donor PBMCs and autologous EBV-transformed B-cell lines transduced with Ad5f35-CMVpp65 chimeric vector	5 × 10^6^ to 1 × 10^8^/m^2^	10/11 remained CMV antigen and DNA negative for mean of 8.3 months. 1 non-evaluable	None	(46)
2008	CMV-specific polyclonal T-cells	12	DCs infected with CMV pp65 protein encoded in adenoviral vector	2 × 10^7^/m^2^	12/12 had CMV immune reconstitution with no need for antiviral therapy	GvHD grade II *n* = 2, GvHD grade III *n* = 2	(47)
2013	CMV-specific CD4+ and CD8+ polyclonal T-cells	50	Monocyte derived DCs either pulsed with CMV pp65 peptide *n* = 10 or transduced to express pp65 protein Ad5f35pp65 *n* = 40 and used to stimulate T-cells	2 × 10^7^/m^2^, insufficient expansion in 9 patients median dose in these patients 1.2 × 10^7^/m^2^	Reduction in % that required anti-viral therapy 17 vs. 36%. No reduction in CMV re-activation rates 1/50 death due to CMV	Acute GvHD grade II-IV *n* = 12, III-IV *n* = 4 Chronic GvHD *n* = 21	(48)
2013	CMV, EBV and Adv-trivirus directed CD4+ and CD8+ polyclonal T-cells	10	DCs nucleofected with DNA plasmids encoding CMV,EBV and Adv viral antigens used to activate T-cells	0.5–2 × 10^7^/m^2^	Off 10, 3 patients had CMV reactivation and 2 patients had CMV/Adv dual infections 4/5 complete CMV clearance 1/5 persistent CMV	GvHD grade I *n* = 1	(49)
**MATCHED DONOR CMV T-CELL LINES (DIRECT SELECTION- MULTIMERS)**							
2005	CMV-specific CD8+ T-cells	9	HLA-peptide tetramer-based selection of CMV-specific CD8+ T-cells	1.2 × 10^3^ to 3.3 × 10^4^/kg	8/9 cleared CMV infection	GvHD grade I *n* = 1, GvHD grade II *n* = 2	(50)
2017	CMV-specific CD8+ T-cells	16	Streptamer HLA-A2 restricted NLV selected	6.3 × 10^6^ cells (HSCT donors) 1.4 × 10^7^ cells (third-part donors)	HSCT 7/7 responded 5/8 third-party responded	GvHD grade II-III *n* = 2	(51)
**MATCHED DONOR CMV T-CELL LINES (DIRECT SELECTION-IFNγ** **CATCH)**							
2010	CMV-specific CD8+ and CD4+ polyclonal T-cells	18	Donor-derived PBMCs stimulated with pp65 protein for 16hrs followed by IFN-γ capture	21 × 10^3^/kg mean dose	15/18 partial or complete CMV viral clearance	GvHD *n* = 1	(52)
2011	CMV-specific CD8+ and CD4+ polyclonal T-cells	18	Donor-derived PBMCs stimulated with pp65 recombinant protein or overlapping peptide pools followed by IFN-γ capture	Target dose 1 × 10^4^ CD3+ T-cells/kg	7/7 prophylactically treated did not have CMV reactivation, 11/11 pre-emptively treated	GvHD grade II *n* = 2, GvHD grade III *n* = 1	(53)
2012	CMV-specific CD4+ and CD8+ polyclonal T-cells	6	Stimulated with peptide followed by IFN-γ capture and culture with autologous feeder cells	6 × 10^5^ to 17 × 10^6^ of 54–96% CMV-specific CD8+ T-cells	6/6 had cleared viremia	None	(54)
**THIRD PARTY CMV T-CELL LINES**							
2013	CMV, EBV, Adv-specific CD4+ and CD8+ polyclonal T-cells	50	PBMCs and autologous EBV-transformed lymphoblast cell lines transduced with Ad5f35-CMVpp65 chimeric vector	2 × 10^7^/m^2^	19/50 treated for persistent CMV and evaluable for response 9/19 CR 8/19 PR 2/19 No response	GvHD grade I *n* = 6, grade II *n* = 1, grade III *n* = 1	(55)
2017	CMV, EBV or Adv mono-valent polyclonal T-cells	30	T-cells stimulated with monocyte derived DCs pulsed with overlapping pepTivators	2 × 10^7^/m^2^ upon persistent viral replication does increased up to 5 × 10^7^/m^2^	28 treated for CMV reactivation 23/30 patients had complete virological response at median 59 days 14/23 patients remained virus PCR negative at median of 326 days	GvHD grade II and IV *n* = 2	(56)
2017	CMV, EBV, Adv, BK virus (BKV) and Human herpesvirus 6 (HHV-6)-specific polyclonal T-cells	37	PBMCs pulsed with pepmix spanning a variety of antigens.	2 × 10^7^/m^2^	17 patients received VST for persistent CMV 6/17 complete responses 10/17 partial responses	GvHD grade III *n* = 1, grade I–II *n* = 5	(57)
2018	CMV-specific polyclonal T-cells	3	Virus-specific T-cell separation (CMV pp65 pepTivator) program by CliniMACS Prodigy Cytokine Capture System	7.5–16.2 × 10^4^ CMV+ T-cell clones/kg	2/3 had viral clearance, 1/3 decrease of viral load	None	(58)
2019	CMV, EBV, and Adv-specific CB derived polyclonal T-cells	14	CB derived DCs transduced with Ad5f35-pp65 antigen used to stimulate CB T-cells (ACT-CAT) *n* = 9 CB derived DCs were stimulated with PepMix containing overlapping peptides for CMV, EBV and Adv antigens used to stimulate CB T-cells (ACT-CAT2)	Dose escalation (ACT-CAT) 2/9 5 × 10^6^/m^2^ 2/9 1 × 10^7^/m^2^ 2/9 1.5 × 10^7^/m^2^ 3/9 2.5 × 10^7^/m^2^ (ACT-CAT2) 5 × 10^6^/m^2^ first dose followed by 1 × 10^7^/m^2^ dose escalation	4/14 had CMV viremia, 1/4 CMV resolution, 2/4 × 1 resolution post-valganciclovir and × 1 resolution post-ganciclovir + x2 additional CB-VST infusions, 1/4 viremia resolved at 6 months but developed CMV retinitis 7/14 treated prophylactically, 6/7 no reactivation, 1/7 Adv reactivation	GVHD *n* = 6	(59)

This review discusses the different approaches required for the commonly encountered post-HSCT CMV clinical scenarios: (1) where the HSCT-donor is CMV seropositive and can thus act as a CMV-specific T-cell donor; and (2) where the HSCT-donor is CMV seronegative and an alternative CMV-specific T-cell source must be sought.

Consideration will also be given to the challenges identified in clinical studies of VSTs to date, such as the potential impact of prolonged *in vitro* culture and the impact of technological advances to optimize VST product purity to reduce the burden of non-viral, potentially alloreactive clones. We will discuss how the VST field has improved outcomes for many patients with life threatening viral infection following HSCT and explore how to broaden the application of CMV VSTs beyond the “patient specific” label.

## Patients With CMV Seropositive Donors

**Figure 1:**
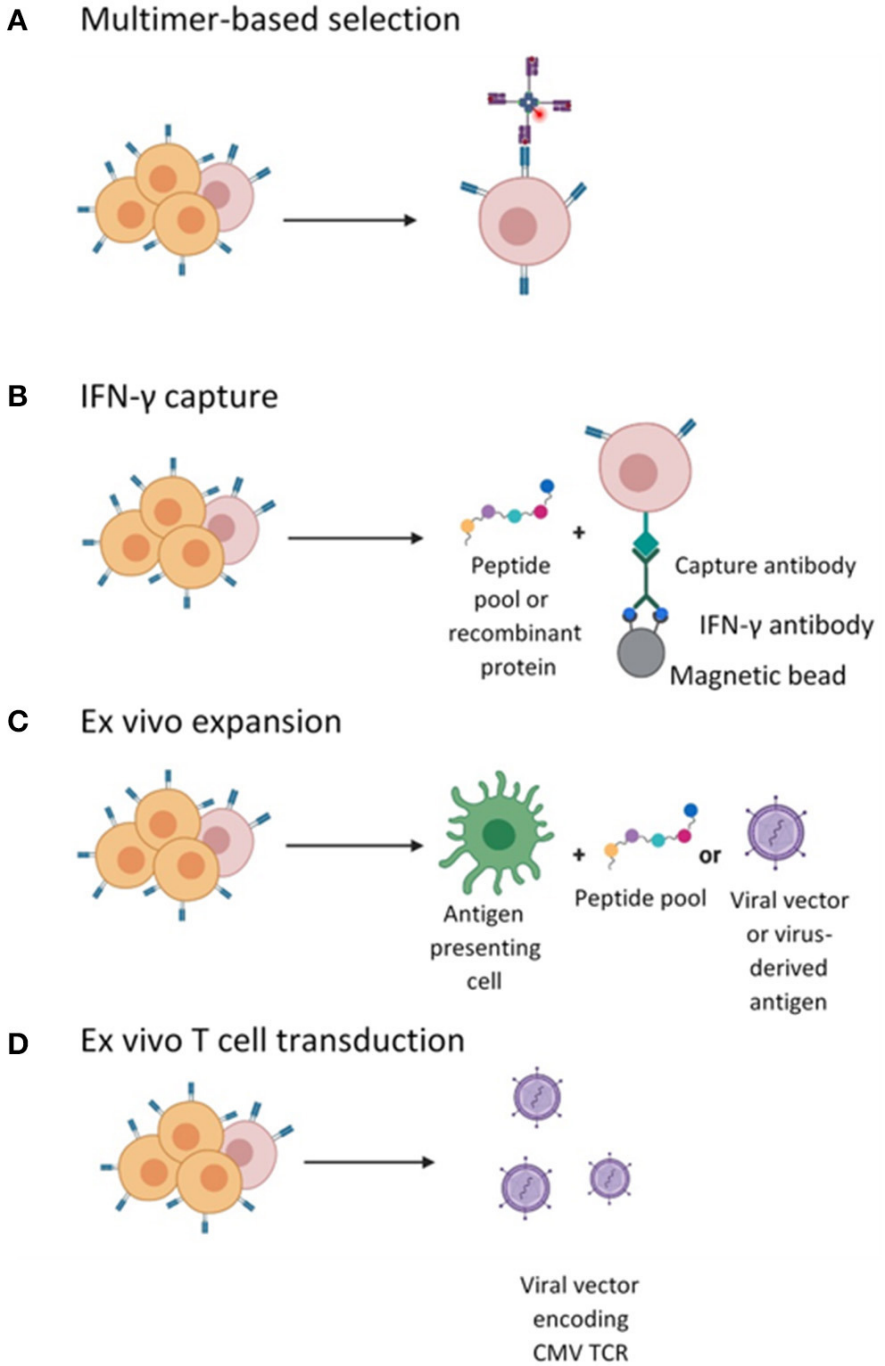
Different strategies employed for the isolation or generation of CMV-specific T cells. **(A)** CMV-specific T cell in the peripheral blood mononuclear cells (PBMCs) are labeled with pMHC I- multimers conjugated to a magnetic bead enabling enrichment of CMV-specific CD8+ CTLs. **(B)** CMV-specific T cells enriched by magnetic selection following stimulation with peptide and IFN-γ secretion and selection. **(C)** *Ex vivo* cell culturing of CMV-specific T cells by stimulation with APCs pulsed with viral peptide or infected with vector encoding viral antigens and expanded in the presence of cytokines. **(D)** *Ex vivo* T cell transduction with lentiviral or retroviral vector encoding a recombinant CMV TCR followed by expansion in the presence of cytokines.

Current evidence suggests a clear relationship between the magnitude of CD8+ T cell responses post-HSCT and CMV viral clearance (60, 61). Indeed, CMV reactivation in patients post-HSCT with seropositive donors is often due to insufficient circulating CMV-specific T-cells due to both conditioning chemotherapy and immunosuppression. Investigators have focussed on whether this can be overcome by the adoptive transfer of CMV-specific T-cells obtained from the matched donor and expanded *ex vivo*. The endpoints for CMV cell therapy studies are often feasibility, toxicity (namely the risk of alloreactive events/GvHD) and efficacy. To date, evidence suggests that adoptive transfer of CMV-specific T cells can reduce the risk of CMV infection and subsequently restore CMV immunity after HSCT in 70–90% of patients with reduced need for antivirals (36). There are several different approaches to the generation of these products, outlined in detail below (and in Figure 1).

### CMV T-Cell Clones

Early studies conducted by Riddell and Walter employed monoclonal selection (by limiting dilution) of CMV-specific CD8+ T-cell clones followed by repeated stimulation with CMV-infected fibroblasts to promote the selective expansion of CMV-specific T-cells. In patients, even at low cell numbers, these products were shown to expand *in vivo* leading to CMV-specific immune reconstitution with persistence up to 8 weeks post-transfer, whilst conferring a low risk of GvHD. Criticisms of this work include the fact that eligibility was limited to patients with sibling donors and that the majority of recruited patients were CMV seronegative, representing a lower risk cohort for CMV disease. Further, criticisms of the manufacture method included both the requirement for viral particles within the culture which portends a risk of viral transfer to the patient and the extensive culture period (8–10 weeks) required to achieve appropriate cell numbers for adoptive transfer (40, 62–64). Despite this, studies of CMV T-cell clones significantly advanced the field: this work not only demonstrated the feasibility of the manufacturing of VSTs, but interestingly showed that patients with CD4+ CMV-specific T-cell populations achieved a more sustained CD8+ T-cell CMV-specific response compared to patients lacking CD4+ T-cells. These studies established a precedent for CMV-specific T cell transfer as a potential immunotherapy for CMV post-HSCT.

### CMV Specific T-Cell Lines

An alternative to CMV T-cell clones is the use of poly or oligo-clonal CMV T-cell lines. These products are enriched for CMV reactivity be expanding on dendritic cells (DCs) pulsed with either CMV lysate or CMV peptides over a short culture period. Peggs et al. took this approach to the clinic. They showed that co-culture of donor-derived PBMCs with CMV-lysate pulsed DCs is a feasible approach to generate products for HSCT patients with detectable blood levels of CMV DNA. These products were infused pre-emptively into CMV seropositive patients who had undergone HSCT (90% of grafts were T cell-depleted). Despite low cell doses, a 3- to 5-fold expansion of the cells was observed *in vivo* within days of adoptive transfer. Furthermore, 50% of patients cleared CMV from the blood with no need for adjuvant antiviral therapy (42). Following this landmark study, the investigators subsequently evaluated the same approach in a larger cohort and were able to demonstrate consistent and durable protective immunity in patients with a reduction in the incidence of secondary CMV infection (65). In the prophylactic setting, a study of CMV-specific T-cell lines administered 29 days following HSCT showed that 22% of all treated patients (2/9) developed CMV infection, but that none of these cases developed into CMV disease and none required antiviral pharmacotherapy. As a biomarker for efficacy, 66% (6/9) of patients exhibited short-lived CMV-specific T-cell engraftment detectable in the blood (66).

There is debate as to the optimal delivery of peptide pulsing in this manufacture setting. The immunodominant CMV-associated pp65 and IE1 epitopes are commonly used and there is most experience with NLVPMVATV (NLV), an HLA-A2 restricted epitope of the pp65 antigen. The criticism of using a single peptide approach is that it delivers monospecific targeting i.e., adoptive immunity is conferred is to a single viral epitope, with the attendant risks of immune escape. Further, by targeting NLV, application of this technology is limited to HLA-A2 patients/donors. To overcome this, several groups have generated multi-antigen targeting “poly-specific” products, by incubating allogeneic T cells *in vitro* with pools of 15-mer peptides spanning the whole pp65 antigen to generate oligoclonal CMV- specific T cells. In clinical studies, poly-specific CMV T cells have been shown to clear CMV viremia, with oligoclonal CMV T cells persisting for up to 2 years in some cases (44, 45).

Gene engineering approaches can also be used for the generation of polyspecific CD4+ and CD8+ CMV T cell lines. Adenoviral vectors encoding whole CMV NLV-derived pp65 antigen can be used to transduce dendritic cells (DCs) to mediate intracellular processing of the pp65 protein and to enable presentation of a variety of pp65 epitopes. CMV-specific T-cells generated in this manner have been evaluated in several clinical studies (46–49). Blyth et al. used matched sibling or closely matched unrelated donor T-cells as starting material. Patients receiving this product were compared to a matched cohort in whom CMV-specific T cells were not administered. The progression-free survival, overall survival, and CMV reactivation incidences were not significantly different between the groups, but in the treated arm there was a reduction in the number of patients requiring antiviral pharmacotherapy for CMV and a 13% reduction in late CMV reactivation (48). Furthermore, there did not appear to be a significant spike in GvHD risk. This manufacturing approach has been shown to generate excellent *in vivo* expansion following infusion of low numbers of poly-specific CMV T cells. Micklethwaite et al. conducted a study of the prophylactic infusion of CMV specific T cells generated from DCs transduced with a CMV pp65 protein-encoding adenoviral vector. They reported no adverse events in any of the 12 adult patients and no need for antiviral pharmacotherapy following the infusion. Additionally, immune reconstitution was observed in all with a predominant increase in CMV-pp65 specific immunity (47). These studies together add to the growing evidence for gene engineering approaches utilizing professional antigen presentation.

### Lessons Learned From Early Experience With CMV Specific T-Cell Manufacturing

Variable study design, dosing, and patient selection/eligibility between reported studies make it challenging to directly compare efficacy and toxicity of donor derived CMV-specific T-cells generated by different methods. However, what is clear from the listed studies is that there is variability in the timelines and perhaps also qualitative differences between the therapeutic products generated. A major challenge in the manufacture of CMV-specific T-cells is the chronic antigen exposure of the harvested T-cells, potentiated by culture conditions requiring repeated exposure to antigen which can lead to features of T-cell exhaustion. Manufacturing is associated with memory inflation of CD8+ CMV-specific populations, but there is commonly also enrichment for terminally differentiated effector T-cells (KLGR1^high^, CD57^high^, CD28^low^, CD27^low^, and CD62L^low^) (50, 67) which have a reduced half-life and a lower proliferative capacity. These characteristics may lead to only transient immune protection in the recipient. In line with developments in manufacturing science for other T-cell products, it is likely that CMV-specific T-cell therapies may benefit from shorter *ex-vivo* expansion to mitigate for the generation of a terminally differentiated products.

CMV T-cell lines have some potential advantages over CMV T-cell clones. Practically, the manufacture process is shorter (CMV T-cell clones can take up to 8–12 weeks) and the potential reduction in vein to vein time may be beneficial for patients. It is not clear whether culture of CMV-reactive T-cells with single peptides vs. overlapping peptide pools vs. proteins vs. gene-engineered professional APCs delivers a superior CMV-specific T-cell product. Virus-free approaches are particularly desirable from a safety perspective, but there may be a trade-off between enhanced safety at the expense of a potentially broader immune reactivity generated with gene-engineered APCs. The importance of this balance remains unproven and studies suggest that even relatively oligoclonal products appear to provide clinical benefit (3). However, controlled clinical trials would be required for a definitive conclusion.

Given the diversity of T-cell responses to CMV described in healthy subjects (3), the future of CMV T-cell line manufacturing is likely to incorporate multi-epitope stimulation strategies across a range of HLA types to generate a more inclusive therapy, broadening access beyond HLA-A2 and to extend anti-viral reactivity beyond a single/few epitope(s) to enhance CMV immune reconstitution.

### Directly Selected CMV T-Cell Products

Initiatives to reduce the requirement for protracted *in vitro* VST culture has prompted the investigation of high stringency CMV T-cell isolation methods. Direct selection by human leucocyte antigen (HLA)-multimers or IFN-γ capture technologies can be used to isolate circulating CMV-specific T-cells from donors with relatively high purity.

### Multimers

HLA class I multimers can be used to select CD8+ CMV-specific T-cell repertoires targeting a single viral epitope through binding of the cognate T-cell receptor (TCR) to HLA monomers loaded with viral peptide (68). Cobbold et al. used HLA-restricted tetramers to isolate TCRs specific to the CMV pp65 and IE1 viral epitopes via magnetic bead-based selection. They demonstrated that tetramer technology is technically feasible at scale: T-cells from CMV seropositive donors can be enriched for CMV-specificity (0.41–12.3% in pre-selection material to 97.8–99.9% following selection) and retain functional activity *in vitro*. In a clinical trial, 9 patients were treated pre-emptively with multimer-selected CD8+ CMV-specific T-cells at a dose of 8.6 × 10^3^/kg and a purity of 98%. Engraftment was observed in all patients up to 10 days post-infusion, with long term persistence reported in 2 patients. All patients had a reduction in CMV viremia and in 8/9 patients there was complete clearance (69).

There are currently many variations on HLA-multimer design, utilizing up to 10 multimerized HLA-monomers for antigen-specific cell selection. Streptamer® is an attractive multimeric selection tool as it delivers competitive reversibility of binding. Indeed, upon exposure to biotin, Streptamer HLA-monomers are released off bound TCR ensuring that Streptamer® is a “non-ATMP” technology by virtue of its removal prior to infusion to the patient. Schmitt et al. isolated CMV-specific CD8+ T cells using Streptamer® for the adoptive transfer to two patients, both of whom demonstrated CMV immune reconstitution (clearance of viral load and Streptamer®-based detection of donor derived CD8+ CMV reactive T cells) (70). Neuenhahn et al. assessed the safety and efficacy of Streptamer®-selected CMV T-cells derived from matched or third-party donors in a phase I/II trial. Sixteen HSCT recipients with drug-refractory CMV infection/reactivation were infused with a single dose of Streptamer®-selected, CMV-specific T-cells isolated from transplant donor (*n* = 8) or third-party donors that were partially matched (*n* = 8). Significant response rates were observed in patients who received matched donor T-cells (51), but lesser responses were observed in recipients of third party products. These studies demonstrate the feasibility and potential antiviral efficacy of products generated using multimer-based isolation. Potential limitations of the multimer method include the lack of available class II multimers for CD4+ CMV-specific T-cell isolation, such that the provision of CD4 T-cell “help” is not yet feasible for these products. Additionally, multimer-based selection has only limited applicability in a subset of HLA types for which multimers are commercially available. For this reason, efforts to develop methods to select polyclonal CMV-T cell products in a non-HLA restricted manner have gained traction. A simple method using IFN-γ capture is attractive for this purpose.

### Interferon Gamma (IFN-γ)-capture

The isolation of CMV-reactive CD4+ and CD8+ T-cells is made possible by stimulation of donor peripheral blood mononucleated cells (PBMCs) with a selection of viral peptides, generating responses against multiple viral epitopes. The stimulated cells secrete IFN-γ and are then captured by an IFN-γ-directed immunomagnetic bead-based platform irrespective of donor HLA type. Using this method, Feuchtinger et al. stimulated donor PBMCs with CMV pp65 peptide followed by an enrichment step for IFN-γ. The cell composition achieved was ~2:1 CMV-reactive CD4:CD8 and the mean total dose achieved was 21 × 10^3^/kg. In 15/18 treated patients, a ≥1 log reduction in blood CMV DNA which observed and was associated with *in vivo* expansion of the transferred cells without an increase in GvHD or acute infusion reactions (52). A follow-on study by Peggs et al. using IFN-γ captured cells as prophylactic or pre-emptive therapy, showed that incubation of (sibling) donor-derived PBMCs with overlapping peptide pools derived from pp65 or recombinant pp65 protein resulted in an improved yield and purity of CMV-specific CD4+ and CD8+ T-cells. The phenotype of the adoptively transferred cells was mainly effector memory with a small fraction of central memory T-cells. Expansion of CD4+ and CD8+ CMV-specific cells was observed *in vivo* within days of transfer. Indeed, the rapid expansion of CMV specific CD8+ cells was associated with an initial increase in central memory populations followed by a secondary expansion of both effector and central memory populations that coincided with a reduction in viremia (53). Following cell dosing, patients in the pre-emptive arm required only 1 antiviral treatment while no patients in the prophylactic arm required antivirals in the ensuing 6 months. This study suggests that product phenotype and the presence of central memory populations may be important for reconstitution of antiviral immunity and clinical outcomes. This merits further investigation and development (71).

Next generation approaches to IFN-γ capture for VST generation include the use of the closed, semi-automated CliniMacs Prodigy® manufacturing platform. Recently Kállay et al. generated VST (EBV, CMV, and AdV) using peptide stimulation followed by IFN-γ capture on the CliniMacs Prodigy Cytokine Capture System (CCS). The resulting cord blood-derived products were infused into 9 pediatric HSCT patients with viral reactivation (including CMV) and 6 of 9 infused patients cleared their viral illness without GvHD, graft rejection or organ toxicity, suggesting that the Prodigy is fast, safe, and effective in VST manufacture (58).

### Lessons Learned From Early Experience With Direct Selection Technologies

It is desirable for the field to find creative solutions for the protracted *in vitro* VST cultures associated with CMV T-cell clones and -lines. For this reason, high stringency (HLA)-multimers or IFN-γ capture are an attractive option. Both methods have shown proof-of-principle in Phase I clinical studies and advantages include a shortened manufacture process and a highly selected (and potentially less alloreactive) product for patients. The potential disadvantages of multimers in this setting mainly relates to their limited availability beyond a narrow range of HLA subtypes and the current lack of available class II multimers for CD4+ CMV-specific T-cell isolation. Furthermore, cGMP CMV Streptamers® are no longer commercially available, making the generation of products for patients very challenging. IFN-γ capture holds several distinct advantages over multimers in that a polyclonal mixed population of CD4 and CD8 T-cells is obtained and that multivirus-specific cells can be easily generated from a single incubation with a range of different viral peptides. Further, the technology is fully scalable to the clean room and can be incorporated onto the CliniMACS Prodigy closed manufacture system. Early data reports viral clearance in some patients infused with VST manufactured in this way but further clinical data will be required to confirm early findings.

## Patients With CMV Seronegative Donors

For patients with seronegative donors, there is a drive to explore third party products and gene engineering strategies to make CMV-reactive T-cell therapies available.

### Third Party VST

For over a decade, personalized CMV-specific T-cell products have been tested in the clinic, but limitations on broad application relate to prolonged manufacturing protocols, high labor costs, and the (lack of) suitable donors. To address this, several groups have generated third-party VST cell banks to create “off-the-shelf” products for immediate use, derived from allogeneic unmatched or HLA-matched sources. The heterogeneity of third-party VST therapy offers the potential advantage of targeting multiple viral epitopes rather than a monospecific approach, potentially enhancing the likelihood of antiviral efficacy. There are concerns around GvHD risk using a third-party approach, as the infused cell product will be mismatched at one or more HLA alleles. Indeed, a high degree of mismatch could lead to host rejection of the infused cells and re-emergent viral activity.

Despite these concerns, several clinical studies have been performed and have shown promising outcomes. Doubrovina et al. observed that 4/5 HSCT patients with Epstein-Barr virus (EBV)-driven post-transplant lymphoproliferative disorder treated with third-party EBV-specific T-cells achieved complete responses without GvHD (72). This approach has also shown applicability in management of CMV and Adenovirus (AdV). Leen et al. generated 32 tri-virus specific T-cell lines against EBV, CMV, and AdV to treat drug-refractory viral infections. Products were selected based on best HLA match and anti-viral activity through the shared allele(s). Despite low overall levels of HLA matching, the *in vivo* safety and complete (CR) or partial response (PR) profiles were compelling. Seventy-four percent of patients achieved CR or PR and durable, ongoing responses were reported in 89% of patients (46, 55, 72).

Despite concerns re mismatch at HLA and the attendant risks of rejection, persistence of third-party VST has been reported for as long as 90 days post-transfer in HSCT recipients (73). Research efforts are underway to determine methods to prolong engraftment of third party VST. Indeed, several groups are focussing on the delivery of polyclonal products comprising CD4+ and CD8+ populations, but this approach requires further optimization (74).

### Third Party VST Banks

Banks of third-party donor derived VST with suitable HLA diversity have been developed to enable ease of HLA allele selection. Withers et al. have reported the safety and efficacy of banked third-party monovalent VSTs generated from 31 donors following infusion into partially matched, heavily pre-treated patients with CMV reactivation. To generate the bank, the authors pulsed donor monocyte derived dendritic cells (MoDCs) with overlapping cGMP PepTivators (Miltenyi Biotec) for CMV, AdV, or EBV. Irradiated peptide-pulsed MoDCs were then co-incubated with donor apheresis/blood for up to 21 days in IL-2 rich medium with a second restimulation with MoDCs at day 7. Despite the infusion of unmatched, unrelated third party CMV T-cells, no GvHD was observed in treated patients and overall responses were reported in 93% of patients at 12 months with complete responses in 76% at 12 months (56).

A third-party VST bank at Memorial Sloan Kettering Cancer Centre (MSKCC) has been established to deliver pp65 reactive CMV-specific T-cell products for patients. This bank has products available for 93% of all HLA-non-identical HSCT recipients and 98% of cord blood recipients (75–77). By comparison, when using HLA-restricted HSCT grafts as the VST source, only 60–70% of patients have an available donor, so a 3rd party bank serves a clinically unmet need (72, 78).

### Cord Blood-Derived Third Party VST

Cord blood-derived VSTs can also be used to generate multi-virus specific T-cells (79). Abraham et al. recently described an approach where a 20% fraction of cord blood units allocated to each cord blood HSCT recipient could be expanded *in vitro* short term (2 weeks) to generate VSTs. Following administration to pediatric cord-blood HSCT patients, they demonstrated safety, feasibility, and persistence. Eighteen out of twenty-one products were successfully manufactured for clinical use and administered products were shown to confer antiviral immunity and/or complement pharmacotherapies. Obstacles still remain in terms of manufacturing times and yield, which is currently being assessed in a follow up clinical trial (NCT03594981) (59).

### Genetic Engineering Approaches

In the setting of a virus naïve donor, it is possible to use genetic engineering to introduce a CMV-specific TCR into polyclonal donor T-cells, harvested by non-mobilized leukapheresis, using viral transduction techniques. The resulting polyclonal T-cell product has engineered specificity for a single CMV epitope. *In vitro* and *in vivo*, CMV-TCR T-cells have preserved functionality, secreting cytokine, and becoming cytotoxic when exposed to CMV-peptide pulsed antigen presenting cells (80–82). Schub et al. isolated the alpha and beta chains from the TCRs of four CMV-specific CD8+ T-cell clones and used retroviral vectors encoding the CMV-specific alpha and beta chains to transduce donor T-cells. The resulting transgenic T-cells were capable of expansion and antigen-specific cytotoxicity *in vivo* in a preclinical model (82). One potential limitation of transgenic TCR immunotherapy is the risk of mispairing of the recombinant and endogenous TCR chains leading to the generation of TCRs with unknown specificity. Several groups are investigating strategies to limit mispairing such as the murinization of the recombinant TCR constant domains and the engineering of recombinant alpha and beta chains to generate an additional disulphide bond (83). More recent developments show that membrane expression of transgenic TCRs can be enhanced by substitution of specific amino acid residues in the framework region of the variable chains. This manipulation resulted in increased proliferation, cytokine production and antigen-specific cytotoxicity of the transgenic TCR T-cells in the presence of low peptide concentrations (84). This is a compelling development and brings transgenic TCR T-cell for viral infections a step closer to clinical application. However, as is the case for any monospecific approach, the limitations here include limited access to patients with specific HLA types and the epitope restriction due to the monoclonal TCR which potentially increases the risk of immune escape downstream.

## Other Compelling Developments

### VSTs Resistant to Immunosuppression

CMV-specific T-cells can restore antiviral immunity with minimal toxicity/GvHD and responses can be durable. However, patients with active GvHD are excluded from studies due to the perceived deleterious impact of immunosuppressive therapy and high-dose corticosteroid on T-cell engraftment and function. In fact, these patients are most likely to experience viral reactivation and represent a group with unmet need in this space. For this reason, several groups are exploring viral T-cells with engineered resistance to immunosuppressants to determine the safety and efficacy of this approach in patients with GvHD requiring immunosuppression. De Angelis et al. generated EBV-cytotoxic lymphocytes (EBV-CTLs) with induced resistance to the immunosuppressant FK506 by knock-down of the FK5062-binding protein (FKBP12). In mice, the resulting EBV-CTLs were resistant to the deleterious effects of FK506 whilst retaining proliferative and cytotoxic functionality (85). In a similar preclinical study, Brewin et al. mutated calcineurin, a key regulator of T cell activation, to disrupt its docking to FK506-FKBP12 and cyclosporin A-cyclophilin A, rendering T-cells resistant to calcineurin inhibitors or FK506 (86). Menger et al. used transcription activator-like effector nucleases (TALEN) genome editing technology to engineer CMV-specific T-cells to be resistant to corticosteroids by inactivating the glucocorticoid receptor (GR). In normal T-cells exposed to corticosteroids, the GR forms a cytosolic complex comprising GR, heat shock protein 70 and 90 and FK506 binding protein which translocates to the nucleus and triggers apoptosis. The authors use Streptamer® technology to isolate CD8+ CMV-specific T-cells from seropositive donors followed by feeder layer expansion and TALEN-mediated inactivation of the GR conferring resistance to steroids. Xenogeneic GvHD models demonstrated the TALEN-edited T-cells to be resistant to corticosteroid-mediated apoptosis *in vivo*. They provided a proof of concept for the development of a clinical protocol for the generation of steroid resistant CTLs for clinical administration (87). More recently, a CRISPR/Cas9-based editing study has demonstrated the feasibility of using this new editing tool to develop steroid resistant CMV-specific T cells for use in a clinical study is underway (77).

### Naive T-Cell Depletion

Preclinical studies suggest that depletion of naïve T-cells from donor lymphocytes (DLI) may reduce the risk of GvHD whilst preserving antiviral immunity. CD62L and CD45RA can be found on the cell surface of naïve T cells and together with other markers such as CD45RO, CCR7, CD27, and CD95 are used to distinguish naïve (Tn), central memory (Tcm), and effector memory T-cell populations (Tem) and CD45RA-re-expressing effector T-cells (Temra). Verfuerth et al. used immunomagnetic CD62L depletion applied to steady state leukapheresis products and found that the resulting CD62L negative fraction comprised equal numbers of CD4+ and CD8+ Tem and Temra. Further, this CD62L- fraction was enriched for pentamer positive antivirus-specific T-cells (88). This is now being tested in a Phase I clinical trial: CD62L-depleted cells are collected from sibling donors and infused into patients between days 24 and 32 following HSCT with the objective of reconstituting antiviral immunity without creating a spike in GvHD (NCT03836690). Bleakley et al. tested a similar hypothesis using CD45RA depletion technology to deliver naïve T cell depleted grafts to patients with high-risk leukemia. They showed that this step reduced the incidence of chronic GvHD whilst preserving the transfer of functional virus-specific immunity (89, 90). This represents a simple approach to a complex problem and further clinical developments and data in this space are eagerly anticipated.

## Conclusion and Future Perspectives

Despite recent advances in the management of CMV following HSCT, there remain significant unmet needs. Monitoring of CMV DNAemia is a key factor in guiding therapeutic intervention. In this review we have discussed novel antiviral drugs with clinical promise such as prophylactic Letermovir, but we acknowledge potential limitations such as high ongoing drug costs associated with population-wide prophylaxis, drug toxicity, and viral resistance. There are sustained efforts to generate CMV immunotherapies, namely adoptive transfer of VSTs, which have been shown to rapidly reconstitute antiviral immunity in clinical studies of patients post-HSCT both with donor-derived or third-party derived CMV-specific T-cells. A number of platforms for VST manufacture have been developed and the resulting cell products have shown expansion and persistence in patients even at low transferred cell numbers, with no excess of GvHD reported, even with third party products where the theoretical concerns of GvHD are highest (91).

Deeper understanding of the optimal phenotype and functionality of the products is an area of active research. It is evident that the differentiation state of the cells may be critical to their function and persistence. In studies of T-cell biology, Gattinoni et al. identified long-lived human memory T cells within the naïve T-cell compartment that possess increased proliferative and self-renewing capacities following antigenic stimulation (92). These desirable characteristics may benefit VST therapy. Modulation of T-cell memory is an area of increasing interest in the field of cellular immunotherapy and small molecules targeting pathways that regulate memory differentiation such as mTOR, Wnt, and PI3K are currently being investigated for use in T-cell manufacturing (93–95) and could be particularly impactful in the VST field.

Well-designed clinical trials are needed to determine optimal donor source, manufacture platform, dosing and timing of CMV T-cell therapy (prophylactic vs. pre-emptive). Furthermore, to truly demonstrate efficacy, randomized studies vs. standard of care will be required with endpoints relating to CMV clearance rather than toxicity. Critically, to move beyond Phase I, a reproducible, feasible, scalable manufacture method will be required and one of the most exciting developments in the VST manufacture space is the CCS/Prodigy combination. This has the potential to streamline VST production from matched donor and third party cell sources (including cords), but further data is required to confirm feasibility and safety of this approach to VST manufacture in patients.

Ultimately, transition of VSTs from academic centers into the commercial setting is likely to improve access to these therapies and in the future should facilitate the initiation of larger, randomized studies to determine efficacy. The challenge will then be one of health economics to determine where VSTs are positioned in relation to other therapies for CMV.

## Author Contributions

MS and CR wrote the article. VM researched the article and provided Table 1. KP contributed to edited and reviewed the content. All authors contributed to the article and approved the submitted version.

## Conflict of Interest

The authors declare that the research was conducted in the absence of any commercial or financial relationships that could be construed as a potential conflict of interest.
